# 3-(Adamantan-1-yl)-1-[(4-benzyl­piperazin-1-yl)meth­yl]-4-phenyl-1*H*-1,2,4-triazole-5(4*H*)-thione

**DOI:** 10.1107/S160053681105570X

**Published:** 2012-01-11

**Authors:** Ebtehal S. Al-Abdullah, Hanadi H. Asiri, Ali El-Emam, Seik Weng Ng

**Affiliations:** a Department of Pharmaceutical Chemistry, College of Pharmacy, King Saud University, Riyadh 11451, Saudi Arabia; b Department of Chemistry, University of Malaya, 50603 Kuala Lumpur, Malaysia; c Chemistry Department, Faculty of Science, King Abdulaziz University, PO Box 80203 Jeddah, Saudi Arabia

## Abstract

The title mol­ecule, C_30_H_37_N_5_S, displays a chair-shaped piperazine ring, as well as an approximately planar triazole ring [maximum deviation = 0.002 (2) Å] whose phenyl substituent is nearly perpendicular to the mean plane of the five-membered ring [dihedral angle = 80.4 (1)°]. The substit­uents on the piperazine ring occupy equatorial sites. Weak inter­molecular C—H⋯S hydrogen bonding is present in the crystal structure.

## Related literature

For background to 3-(1-adamant­yl)-4-substituted-5-mercapto-1,2,4-triazole derivatives, see: El-Emam & Ibrahim (1991 ▶).
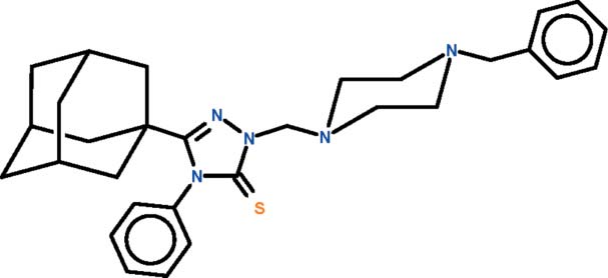


## Experimental

### 

#### Crystal data

C_30_H_37_N_5_S
*M* *_r_* = 499.71
Triclinic, 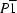

*a* = 10.1677 (6) Å
*b* = 11.3287 (7) Å
*c* = 12.5331 (7) Å
α = 67.037 (6)°
β = 85.768 (5)°
γ = 83.547 (5)°
*V* = 1320.12 (13) Å^3^
*Z* = 2
Mo *K*α radiation
μ = 0.15 mm^−1^
*T* = 100 K
0.15 × 0.15 × 0.05 mm

#### Data collection

Agilent SuperNova Dual diffractometer with an Atlas detector
Absorption correction: multi-scan (*CrysAlis PRO*; Agilent, 2010 ▶) *T*_min_ = 0.978, *T*_max_ = 0.993
9288 measured reflections
6033 independent reflections
4002 reflections with *I* > 2σ(*I*)
*R*_int_ = 0.035

#### Refinement

*R*[*F* ^2^ > 2σ(*F* ^2^)] = 0.064
*wR*(*F* ^2^) = 0.151
*S* = 1.01
6033 reflections
325 parameters
H-atom parameters constrained
Δρ_max_ = 0.76 e Å^−3^
Δρ_min_ = −0.39 e Å^−3^

### 

Data collection: *CrysAlis PRO* (Agilent, 2010 ▶); cell refinement: *CrysAlis PRO*; data reduction: *CrysAlis PRO*; program(s) used to solve structure: *SHELXS97* (Sheldrick, 2008 ▶); program(s) used to refine structure: *SHELXL97* (Sheldrick, 2008 ▶); molecular graphics: *X-SEED* (Barbour, 2001 ▶); software used to prepare material for publication: *publCIF* (Westrip, 2010 ▶).

## Supplementary Material

Crystal structure: contains datablock(s) global, I. DOI: 10.1107/S160053681105570X/xu5419sup1.cif

Structure factors: contains datablock(s) I. DOI: 10.1107/S160053681105570X/xu5419Isup2.hkl

Supplementary material file. DOI: 10.1107/S160053681105570X/xu5419Isup3.cml

Additional supplementary materials:  crystallographic information; 3D view; checkCIF report

## Figures and Tables

**Table 1:** Hydrogen-bond geometry (Å, °)

*D*—H⋯*A*	*D*—H	H⋯*A*	*D*⋯*A*	*D*—H⋯*A*
C13—H13⋯S1^i^	1.00	2.85	3.751 (3)	150
C28—H28⋯S1^ii^	0.95	2.84	3.673 (4)	146

Symmetry codes: (i) 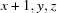; (ii) 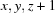.

**Fig. 1.**
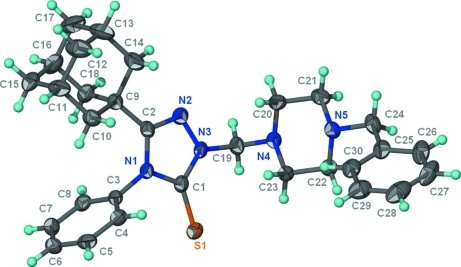
Thermal ellipsoid plot (Barbour, 2001) of C30H37N5S at the 70% probability level; hydrogen atoms are drawn as spheres of arbitrary radius.

## supplementary crystallographic information

### Comment

We reported the synthesis, anti-inflammatory and analgesic properties of 3-(1-adamantyl)-4-substituted-5-mercapto-1,2,4-triazole derivatives (El-Emam & Ibrahim, 1991). The triazole ring, which possesses a secondary nitrogen site next to a double-bond sulfur, is capable of undergoing a Mannich reaction with an *N*-substituted piperazine derivative to yield a new class of chemotherapeutic compounds. The C_30_H_37_N_5_S molecule (Scheme I, Fig. 1) displays a chair-shaped piperazine ring, as well as a planar triazole ring whose phenyl substituent is nearly perpendicular to the mean plane of the five-membered ring (dihedral angle 80.4 (1)°).

### Experimental

5-(1-Adamantyl)-4-phenyl-1,2,4-triazole-3-thiol was synthesized according to a reported procedure (El-Emam & Ibrahim, 1991). The compound (2 mmol), 1-benzylpiperazine (2 mmol) and a 37% formaldehyde solution (0.5 ml) in ethanol (8 ml), was heated for 15 minutes. Stirring was continued for 12 h at room temperature. The product was filtered, washed with water, dried, and recrystallized from ethanol to yield (80%) of the title compound as colorless crystals, m.p. 470–472 K.

### Refinement

Carbon-bound H-atoms were placed in calculated positions [C–H 0.95 to 1.00 Å, *U*_iso_(H) 1.2 to 1.5*U*_eq_(C)] and were included in the refinement in the riding model approximation.

### Crystal data

**Table:**

C_30_H_37_N_5_S	*Z* = 2
*M**_r_* = 499.71	*F*(000) = 536
Triclinic, *P*1	*D*_x_ = 1.257 Mg m^−^^3^
Hall symbol: -P 1	Mo *K*α radiation, λ = 0.71073 Å
*a* = 10.1677 (6) Å	Cell parameters from 2374 reflections
*b* = 11.3287 (7) Å	θ = 2.6–27.5°
*c* = 12.5331 (7) Å	µ = 0.15 mm^−^^1^
α = 67.037 (6)°	*T* = 100 K
β = 85.768 (5)°	Irregular, colorless
γ = 83.547 (5)°	0.15 × 0.15 × 0.05 mm
*V* = 1320.12 (13) Å^3^	

### Data collection

**Table:**

Agilent SuperNova Dual diffractometer with an Atlas detector	6033 independent reflections
Radiation source: SuperNova (Mo) X-ray Source	4002 reflections with *I* > 2σ(*I*)
Mirror	*R*_int_ = 0.035
Detector resolution: 10.4041 pixels mm^-1^	θ_max_ = 27.6°, θ_min_ = 2.6°
ω scan	*h* = −13→13
Absorption correction: multi-scan (CrysAlis PRO; Agilent, 2010)	*k* = −11→14
*T*_min_ = 0.978, *T*_max_ = 0.993	*l* = −13→16
9288 measured reflections	

### Refinement

**Table:**

Refinement on *F*^2^	Primary atom site location: structure-invariant direct methods
Least-squares matrix: full	Secondary atom site location: difference Fourier map
*R*[*F*^2^ > 2σ(*F*^2^)] = 0.064	Hydrogen site location: inferred from neighbouring sites
*wR*(*F*^2^) = 0.151	H-atom parameters constrained
*S* = 1.01	*w* = 1/[σ^2^(*F*_o_^2^) + (0.0475*P*)^2^ + 0.7934*P*] where *P* = (*F*_o_^2^ + 2*F*_c_^2^)/3
6033 reflections	(Δ/σ)_max_ = 0.001
325 parameters	Δρ_max_ = 0.76 e Å^−^^3^
0 restraints	Δρ_min_ = −0.39 e Å^−^^3^

### Fractional atomic coordinates and isotropic or equivalent isotropic displacement parameters (Å^2^)

**Table:**

	*x*	*y*	*z*	*U*_iso_*/*U*_eq_	
S1	0.03136 (7)	0.21542 (6)	0.47877 (6)	0.03032 (19)	
N1	0.2538 (2)	0.34089 (19)	0.38748 (16)	0.0234 (5)	
N2	0.4183 (2)	0.1937 (2)	0.46788 (17)	0.0288 (5)	
N3	0.2963 (2)	0.1453 (2)	0.50537 (17)	0.0266 (5)	
N4	0.3003 (2)	−0.0352 (2)	0.69837 (17)	0.0248 (5)	
N5	0.3255 (2)	−0.0357 (2)	0.92328 (17)	0.0267 (5)	
C1	0.1931 (2)	0.2324 (2)	0.4584 (2)	0.0244 (6)	
C2	0.3904 (2)	0.3133 (2)	0.3963 (2)	0.0241 (6)	
C3	0.1796 (2)	0.4566 (2)	0.3133 (2)	0.0230 (5)	
C4	0.1219 (2)	0.5441 (2)	0.3584 (2)	0.0264 (6)	
H4	0.1265	0.5264	0.4387	0.032*	
C5	0.0571 (2)	0.6581 (2)	0.2848 (2)	0.0296 (6)	
H5	0.0174	0.7196	0.3144	0.036*	
C6	0.0503 (3)	0.6822 (3)	0.1679 (2)	0.0318 (6)	
H6	0.0076	0.7612	0.1173	0.038*	
C7	0.1052 (3)	0.5919 (3)	0.1245 (2)	0.0307 (6)	
H7	0.0989	0.6084	0.0446	0.037*	
C8	0.1691 (2)	0.4778 (2)	0.1973 (2)	0.0251 (6)	
H8	0.2054	0.4146	0.1684	0.030*	
C9	0.4971 (2)	0.3991 (2)	0.3296 (2)	0.0252 (6)	
C10	0.4604 (3)	0.5433 (2)	0.3052 (2)	0.0283 (6)	
H10A	0.3785	0.5737	0.2604	0.034*	
H10B	0.4433	0.5550	0.3794	0.034*	
C11	0.5733 (3)	0.6229 (3)	0.2364 (3)	0.0354 (7)	
H11	0.5476	0.7159	0.2204	0.043*	
C12	0.6989 (3)	0.5787 (3)	0.3071 (3)	0.0475 (8)	
H12A	0.7716	0.6305	0.2633	0.057*	
H12B	0.6827	0.5913	0.3810	0.057*	
C13	0.7381 (3)	0.4360 (3)	0.3320 (3)	0.0444 (8)	
H13	0.8199	0.4068	0.3784	0.053*	
C14	0.6254 (3)	0.3553 (3)	0.4001 (2)	0.0359 (7)	
H14A	0.6086	0.3653	0.4751	0.043*	
H14B	0.6515	0.2633	0.4164	0.043*	
C15	0.5980 (3)	0.6050 (3)	0.1216 (2)	0.0402 (7)	
H15A	0.6685	0.6586	0.0752	0.048*	
H15B	0.5163	0.6329	0.0766	0.048*	
C16	0.6400 (3)	0.4628 (3)	0.1454 (3)	0.0392 (7)	
H16	0.6575	0.4516	0.0702	0.047*	
C17	0.7653 (3)	0.4186 (3)	0.2161 (3)	0.0484 (9)	
H17A	0.8383	0.4700	0.1720	0.058*	
H17B	0.7923	0.3270	0.2311	0.058*	
C18	0.5277 (3)	0.3819 (3)	0.2139 (2)	0.0292 (6)	
H18A	0.5536	0.2900	0.2295	0.035*	
H18B	0.4472	0.4087	0.1672	0.035*	
C19	0.2901 (3)	0.0056 (2)	0.5756 (2)	0.0273 (6)	
H19A	0.2053	−0.0195	0.5606	0.033*	
H19B	0.3623	−0.0424	0.5475	0.033*	
C20	0.4283 (2)	−0.0210 (3)	0.7366 (2)	0.0284 (6)	
H20A	0.4393	0.0713	0.7148	0.034*	
H20B	0.5011	−0.0578	0.6987	0.034*	
C21	0.4328 (3)	−0.0913 (3)	0.8676 (2)	0.0301 (6)	
H21A	0.4232	−0.1838	0.8890	0.036*	
H21B	0.5193	−0.0836	0.8946	0.036*	
C22	0.1978 (2)	−0.0506 (3)	0.8851 (2)	0.0269 (6)	
H22A	0.1249	−0.0140	0.9231	0.032*	
H22B	0.1872	−0.1431	0.9076	0.032*	
C23	0.1912 (2)	0.0185 (2)	0.7543 (2)	0.0263 (6)	
H23A	0.1054	0.0078	0.7282	0.032*	
H23B	0.1980	0.1116	0.7320	0.032*	
C24	0.3334 (3)	−0.0941 (3)	1.0496 (2)	0.0312 (6)	
H24A	0.4273	−0.1060	1.0706	0.037*	
H24B	0.2998	−0.1802	1.0783	0.037*	
C25	0.2551 (3)	−0.0143 (3)	1.1093 (2)	0.0302 (6)	
C26	0.2347 (3)	−0.0657 (3)	1.2300 (2)	0.0394 (7)	
H26	0.2647	−0.1529	1.2735	0.047*	
C27	0.1715 (3)	0.0091 (4)	1.2860 (3)	0.0466 (9)	
H27	0.1591	−0.0270	1.3679	0.056*	
C28	0.1259 (3)	0.1364 (4)	1.2242 (3)	0.0458 (8)	
H28	0.0818	0.1873	1.2633	0.055*	
C29	0.1449 (3)	0.1876 (3)	1.1068 (2)	0.0376 (7)	
H29	0.1141	0.2747	1.0640	0.045*	
C30	0.2092 (3)	0.1134 (3)	1.0491 (2)	0.0304 (6)	
H30	0.2219	0.1507	0.9672	0.036*	

### Atomic displacement parameters (Å^2^)

**Table:**

	*U*^11^	*U*^22^	*U*^33^	*U*^12^	*U*^13^	*U*^23^
S1	0.0259 (4)	0.0258 (4)	0.0312 (4)	−0.0016 (3)	0.0045 (3)	−0.0034 (3)
N1	0.0239 (11)	0.0159 (10)	0.0236 (11)	0.0017 (9)	0.0038 (8)	−0.0019 (8)
N2	0.0271 (12)	0.0223 (12)	0.0291 (12)	0.0024 (9)	0.0050 (9)	−0.0036 (9)
N3	0.0258 (12)	0.0199 (11)	0.0244 (11)	0.0011 (9)	0.0052 (9)	0.0002 (8)
N4	0.0250 (11)	0.0218 (11)	0.0231 (11)	−0.0007 (9)	−0.0002 (8)	−0.0042 (8)
N5	0.0267 (12)	0.0239 (12)	0.0255 (11)	−0.0020 (9)	−0.0037 (9)	−0.0047 (9)
C1	0.0274 (14)	0.0196 (13)	0.0220 (13)	0.0004 (10)	0.0033 (10)	−0.0049 (10)
C2	0.0276 (14)	0.0196 (13)	0.0207 (12)	0.0035 (11)	0.0003 (10)	−0.0045 (10)
C3	0.0204 (13)	0.0156 (12)	0.0265 (13)	−0.0018 (10)	0.0009 (10)	−0.0013 (10)
C4	0.0241 (14)	0.0255 (14)	0.0272 (13)	−0.0030 (11)	0.0023 (10)	−0.0080 (11)
C5	0.0205 (13)	0.0231 (14)	0.0424 (16)	−0.0017 (11)	0.0020 (11)	−0.0103 (12)
C6	0.0240 (14)	0.0210 (14)	0.0383 (16)	−0.0002 (11)	−0.0025 (11)	0.0016 (11)
C7	0.0272 (14)	0.0304 (15)	0.0269 (14)	−0.0069 (12)	−0.0034 (11)	−0.0014 (11)
C8	0.0243 (13)	0.0225 (13)	0.0274 (13)	−0.0069 (11)	0.0030 (10)	−0.0079 (10)
C9	0.0244 (13)	0.0215 (13)	0.0253 (13)	0.0011 (11)	0.0007 (10)	−0.0054 (10)
C10	0.0259 (14)	0.0211 (13)	0.0353 (15)	0.0006 (11)	−0.0033 (11)	−0.0085 (11)
C11	0.0243 (15)	0.0232 (14)	0.0557 (18)	−0.0027 (11)	−0.0055 (12)	−0.0111 (13)
C12	0.0303 (17)	0.0334 (18)	0.077 (2)	−0.0034 (13)	−0.0110 (15)	−0.0177 (16)
C13	0.0258 (16)	0.0371 (18)	0.065 (2)	0.0036 (13)	−0.0158 (14)	−0.0134 (15)
C14	0.0317 (16)	0.0301 (16)	0.0410 (16)	0.0061 (13)	−0.0090 (12)	−0.0095 (12)
C15	0.0304 (16)	0.0332 (17)	0.0441 (17)	−0.0103 (13)	0.0074 (13)	−0.0004 (13)
C16	0.0340 (16)	0.0376 (17)	0.0411 (17)	−0.0087 (13)	0.0128 (13)	−0.0110 (13)
C17	0.0266 (16)	0.0361 (18)	0.076 (2)	−0.0025 (14)	0.0127 (15)	−0.0165 (16)
C18	0.0298 (15)	0.0251 (14)	0.0302 (14)	−0.0057 (11)	0.0057 (11)	−0.0082 (11)
C19	0.0310 (15)	0.0171 (13)	0.0264 (13)	0.0028 (11)	0.0024 (11)	−0.0024 (10)
C20	0.0213 (13)	0.0231 (14)	0.0354 (15)	0.0000 (11)	0.0008 (11)	−0.0064 (11)
C21	0.0254 (14)	0.0243 (14)	0.0381 (15)	0.0003 (11)	−0.0051 (11)	−0.0092 (11)
C22	0.0248 (14)	0.0258 (14)	0.0277 (14)	−0.0050 (11)	−0.0016 (10)	−0.0069 (11)
C23	0.0215 (13)	0.0261 (14)	0.0264 (13)	−0.0022 (11)	0.0001 (10)	−0.0050 (10)
C24	0.0376 (16)	0.0224 (14)	0.0284 (14)	−0.0054 (12)	−0.0082 (11)	−0.0022 (11)
C25	0.0299 (15)	0.0330 (16)	0.0275 (14)	−0.0146 (12)	−0.0025 (11)	−0.0080 (11)
C26	0.0472 (18)	0.0422 (18)	0.0277 (15)	−0.0247 (15)	−0.0038 (13)	−0.0062 (13)
C27	0.048 (2)	0.070 (2)	0.0283 (16)	−0.0365 (18)	0.0087 (13)	−0.0200 (16)
C28	0.0383 (18)	0.064 (2)	0.0495 (19)	−0.0237 (16)	0.0098 (14)	−0.0349 (17)
C29	0.0314 (16)	0.0454 (18)	0.0431 (17)	−0.0077 (13)	0.0013 (13)	−0.0241 (14)
C30	0.0309 (15)	0.0328 (15)	0.0292 (14)	−0.0082 (12)	0.0008 (11)	−0.0127 (12)

### Geometric parameters (Å, °)

**Table:**

S1—C1	1.667 (3)	C13—H13	1.0000
N1—C1	1.385 (3)	C14—H14A	0.9900
N1—C2	1.391 (3)	C14—H14B	0.9900
N1—C3	1.443 (3)	C15—C16	1.535 (4)
N2—C2	1.313 (3)	C15—H15A	0.9900
N2—N3	1.388 (3)	C15—H15B	0.9900
N3—C1	1.351 (3)	C16—C17	1.528 (4)
N3—C19	1.487 (3)	C16—C18	1.536 (4)
N4—C19	1.432 (3)	C16—H16	1.0000
N4—C20	1.465 (3)	C17—H17A	0.9900
N4—C23	1.469 (3)	C17—H17B	0.9900
N5—C22	1.466 (3)	C18—H18A	0.9900
N5—C21	1.467 (3)	C18—H18B	0.9900
N5—C24	1.463 (3)	C19—H19A	0.9900
C2—C9	1.513 (3)	C19—H19B	0.9900
C3—C4	1.380 (3)	C20—C21	1.521 (3)
C3—C8	1.388 (3)	C20—H20A	0.9900
C4—C5	1.387 (3)	C20—H20B	0.9900
C4—H4	0.9500	C21—H21A	0.9900
C5—C6	1.387 (4)	C21—H21B	0.9900
C5—H5	0.9500	C22—C23	1.519 (3)
C6—C7	1.384 (4)	C22—H22A	0.9900
C6—H6	0.9500	C22—H22B	0.9900
C7—C8	1.380 (3)	C23—H23A	0.9900
C7—H7	0.9500	C23—H23B	0.9900
C8—H8	0.9500	C24—C25	1.513 (4)
C9—C18	1.542 (3)	C24—H24A	0.9900
C9—C10	1.547 (3)	C24—H24B	0.9900
C9—C14	1.552 (4)	C25—C30	1.389 (4)
C10—C11	1.535 (4)	C25—C26	1.401 (4)
C10—H10A	0.9900	C26—C27	1.378 (4)
C10—H10B	0.9900	C26—H26	0.9500
C11—C15	1.530 (4)	C27—C28	1.389 (5)
C11—C12	1.530 (4)	C27—H27	0.9500
C11—H11	1.0000	C28—C29	1.362 (4)
C12—C13	1.533 (4)	C28—H28	0.9500
C12—H12A	0.9900	C29—C30	1.393 (4)
C12—H12B	0.9900	C29—H29	0.9500
C13—C14	1.536 (4)	C30—H30	0.9500
C13—C17	1.541 (5)		
			
C1—N1—C2	108.75 (19)	C11—C15—H15B	109.7
C1—N1—C3	122.2 (2)	C16—C15—H15B	109.7
C2—N1—C3	128.9 (2)	H15A—C15—H15B	108.2
C2—N2—N3	104.9 (2)	C17—C16—C18	109.5 (2)
C1—N3—N2	113.09 (19)	C17—C16—C15	110.0 (3)
C1—N3—C19	126.6 (2)	C18—C16—C15	108.9 (2)
N2—N3—C19	119.69 (19)	C17—C16—H16	109.5
C19—N4—C20	115.21 (19)	C18—C16—H16	109.5
C19—N4—C23	114.27 (19)	C15—C16—H16	109.5
C20—N4—C23	110.6 (2)	C16—C17—C13	109.4 (2)
C22—N5—C21	109.4 (2)	C16—C17—H17A	109.8
C22—N5—C24	111.6 (2)	C13—C17—H17A	109.8
C21—N5—C24	111.13 (19)	C16—C17—H17B	109.8
N3—C1—N1	103.3 (2)	C13—C17—H17B	109.8
N3—C1—S1	129.07 (19)	H17A—C17—H17B	108.2
N1—C1—S1	127.64 (18)	C16—C18—C9	110.6 (2)
N2—C2—N1	109.9 (2)	C16—C18—H18A	109.5
N2—C2—C9	122.1 (2)	C9—C18—H18A	109.5
N1—C2—C9	127.8 (2)	C16—C18—H18B	109.5
C4—C3—C8	121.4 (2)	C9—C18—H18B	109.5
C4—C3—N1	119.6 (2)	H18A—C18—H18B	108.1
C8—C3—N1	119.0 (2)	N4—C19—N3	116.7 (2)
C3—C4—C5	119.0 (2)	N4—C19—H19A	108.1
C3—C4—H4	120.5	N3—C19—H19A	108.1
C5—C4—H4	120.5	N4—C19—H19B	108.1
C4—C5—C6	119.9 (2)	N3—C19—H19B	108.1
C4—C5—H5	120.1	H19A—C19—H19B	107.3
C6—C5—H5	120.1	N4—C20—C21	108.8 (2)
C7—C6—C5	120.5 (2)	N4—C20—H20A	109.9
C7—C6—H6	119.8	C21—C20—H20A	109.9
C5—C6—H6	119.8	N4—C20—H20B	109.9
C8—C7—C6	120.0 (2)	C21—C20—H20B	109.9
C8—C7—H7	120.0	H20A—C20—H20B	108.3
C6—C7—H7	120.0	N5—C21—C20	109.7 (2)
C7—C8—C3	119.1 (2)	N5—C21—H21A	109.7
C7—C8—H8	120.4	C20—C21—H21A	109.7
C3—C8—H8	120.4	N5—C21—H21B	109.7
C2—C9—C18	108.7 (2)	C20—C21—H21B	109.7
C2—C9—C10	113.9 (2)	H21A—C21—H21B	108.2
C18—C9—C10	109.4 (2)	N5—C22—C23	109.6 (2)
C2—C9—C14	109.0 (2)	N5—C22—H22A	109.7
C18—C9—C14	107.8 (2)	C23—C22—H22A	109.7
C10—C9—C14	107.9 (2)	N5—C22—H22B	109.7
C11—C10—C9	110.3 (2)	C23—C22—H22B	109.7
C11—C10—H10A	109.6	H22A—C22—H22B	108.2
C9—C10—H10A	109.6	N4—C23—C22	109.52 (19)
C11—C10—H10B	109.6	N4—C23—H23A	109.8
C9—C10—H10B	109.6	C22—C23—H23A	109.8
H10A—C10—H10B	108.1	N4—C23—H23B	109.8
C15—C11—C10	109.1 (2)	C22—C23—H23B	109.8
C15—C11—C12	110.1 (2)	H23A—C23—H23B	108.2
C10—C11—C12	109.6 (2)	N5—C24—C25	113.1 (2)
C15—C11—H11	109.3	N5—C24—H24A	109.0
C10—C11—H11	109.3	C25—C24—H24A	109.0
C12—C11—H11	109.3	N5—C24—H24B	109.0
C13—C12—C11	109.3 (3)	C25—C24—H24B	109.0
C13—C12—H12A	109.8	H24A—C24—H24B	107.8
C11—C12—H12A	109.8	C30—C25—C26	117.8 (3)
C13—C12—H12B	109.8	C30—C25—C24	121.9 (2)
C11—C12—H12B	109.8	C26—C25—C24	120.1 (3)
H12A—C12—H12B	108.3	C27—C26—C25	120.6 (3)
C12—C13—C14	109.9 (2)	C27—C26—H26	119.7
C12—C13—C17	109.1 (3)	C25—C26—H26	119.7
C14—C13—C17	109.4 (3)	C26—C27—C28	120.8 (3)
C12—C13—H13	109.5	C26—C27—H27	119.6
C14—C13—H13	109.5	C28—C27—H27	119.6
C17—C13—H13	109.5	C29—C28—C27	119.2 (3)
C13—C14—C9	110.4 (2)	C29—C28—H28	120.4
C13—C14—H14A	109.6	C27—C28—H28	120.4
C9—C14—H14A	109.6	C28—C29—C30	120.6 (3)
C13—C14—H14B	109.6	C28—C29—H29	119.7
C9—C14—H14B	109.6	C30—C29—H29	119.7
H14A—C14—H14B	108.1	C25—C30—C29	121.0 (3)
C11—C15—C16	109.7 (2)	C25—C30—H30	119.5
C11—C15—H15A	109.7	C29—C30—H30	119.5
C16—C15—H15A	109.7		
			
C2—N2—N3—C1	0.1 (3)	C17—C13—C14—C9	−60.1 (3)
C2—N2—N3—C19	171.5 (2)	C2—C9—C14—C13	177.1 (2)
N2—N3—C1—N1	0.2 (3)	C18—C9—C14—C13	59.3 (3)
C19—N3—C1—N1	−170.6 (2)	C10—C9—C14—C13	−58.8 (3)
N2—N3—C1—S1	179.86 (19)	C10—C11—C15—C16	−61.5 (3)
C19—N3—C1—S1	9.1 (4)	C12—C11—C15—C16	58.9 (3)
C2—N1—C1—N3	−0.3 (3)	C11—C15—C16—C17	−58.7 (3)
C3—N1—C1—N3	175.7 (2)	C11—C15—C16—C18	61.2 (3)
C2—N1—C1—S1	179.95 (19)	C18—C16—C17—C13	−59.9 (3)
C3—N1—C1—S1	−4.0 (4)	C15—C16—C17—C13	59.6 (3)
N3—N2—C2—N1	−0.3 (3)	C12—C13—C17—C16	−60.5 (3)
N3—N2—C2—C9	−176.2 (2)	C14—C13—C17—C16	59.8 (3)
C1—N1—C2—N2	0.4 (3)	C17—C16—C18—C9	60.8 (3)
C3—N1—C2—N2	−175.2 (2)	C15—C16—C18—C9	−59.4 (3)
C1—N1—C2—C9	176.0 (2)	C2—C9—C18—C16	−177.5 (2)
C3—N1—C2—C9	0.3 (4)	C10—C9—C18—C16	57.6 (3)
C1—N1—C3—C4	83.1 (3)	C14—C9—C18—C16	−59.5 (3)
C2—N1—C3—C4	−101.8 (3)	C20—N4—C19—N3	−65.8 (3)
C1—N1—C3—C8	−97.5 (3)	C23—N4—C19—N3	63.8 (3)
C2—N1—C3—C8	77.6 (3)	C1—N3—C19—N4	−103.1 (3)
C8—C3—C4—C5	−2.9 (4)	N2—N3—C19—N4	86.8 (3)
N1—C3—C4—C5	176.5 (2)	C19—N4—C20—C21	−169.2 (2)
C3—C4—C5—C6	0.5 (4)	C23—N4—C20—C21	59.4 (3)
C4—C5—C6—C7	1.5 (4)	C22—N5—C21—C20	60.9 (3)
C5—C6—C7—C8	−1.0 (4)	C24—N5—C21—C20	−175.4 (2)
C6—C7—C8—C3	−1.3 (4)	N4—C20—C21—N5	−60.1 (3)
C4—C3—C8—C7	3.3 (4)	C21—N5—C22—C23	−60.0 (3)
N1—C3—C8—C7	−176.1 (2)	C24—N5—C22—C23	176.6 (2)
N2—C2—C9—C18	88.9 (3)	C19—N4—C23—C22	169.1 (2)
N1—C2—C9—C18	−86.1 (3)	C20—N4—C23—C22	−59.0 (3)
N2—C2—C9—C10	−148.8 (2)	N5—C22—C23—N4	58.8 (3)
N1—C2—C9—C10	36.1 (4)	C22—N5—C24—C25	−75.8 (3)
N2—C2—C9—C14	−28.3 (3)	C21—N5—C24—C25	161.8 (2)
N1—C2—C9—C14	156.6 (2)	N5—C24—C25—C30	−14.8 (4)
C2—C9—C10—C11	−179.4 (2)	N5—C24—C25—C26	169.7 (2)
C18—C9—C10—C11	−57.6 (3)	C30—C25—C26—C27	−0.2 (4)
C14—C9—C10—C11	59.4 (3)	C24—C25—C26—C27	175.5 (3)
C9—C10—C11—C15	59.7 (3)	C25—C26—C27—C28	0.5 (4)
C9—C10—C11—C12	−61.0 (3)	C26—C27—C28—C29	−0.4 (5)
C15—C11—C12—C13	−60.0 (3)	C27—C28—C29—C30	0.1 (4)
C10—C11—C12—C13	60.1 (3)	C26—C25—C30—C29	−0.1 (4)
C11—C12—C13—C14	−59.5 (3)	C24—C25—C30—C29	−175.7 (3)
C11—C12—C13—C17	60.5 (3)	C28—C29—C30—C25	0.2 (4)
C12—C13—C14—C9	59.7 (3)		

### Hydrogen-bond geometry (Å, °)

**Table:**

*D*—H···*A*	*D*—H	H···*A*	*D*···*A*	*D*—H···*A*
C13—H13···S1^i^	1.00	2.85	3.751 (3)	150
C28—H28···S1^ii^	0.95	2.84	3.673 (4)	146

Symmetry codes: (i) *x*+1, *y*, *z*; (ii) *x*, *y*, *z*+1.

## References

[bb1] Agilent (2010). *CrysAlis PRO* Agilent Technologies, Yarnton, England.

[bb2] Barbour, L. J. (2001). *J. Supramol. Chem.* **1**, 189–191.

[bb3] El-Emam, A. A. & Ibrahim, T. M. (1991). *Arzneim. Forsch./Drug Res.* **41**, 1260–1264. 1815527

[bb4] Sheldrick, G. M. (2008). *Acta Cryst.* A**64**, 112–122. 10.1107/S0108767307043930 18156677

[bb5] Westrip, S. P. (2010). *J. Appl. Cryst.* **43**, 920–925.

